# Regulation of Nrf2/Keap1 signalling in human skeletal muscle during exercise to exhaustion in normoxia, severe acute hypoxia and post-exercise ischaemia: Influence of metabolite accumulation and oxygenation

**DOI:** 10.1016/j.redox.2020.101627

**Published:** 2020-06-30

**Authors:** Angel Gallego-Selles, Marcos Martin-Rincon, Miriam Martinez-Canton, Mario Perez-Valera, Saúl Martín-Rodríguez, Miriam Gelabert-Rebato, Alfredo Santana, David Morales-Alamo, Cecilia Dorado, Jose A.L. Calbet

**Affiliations:** a Department of Physical Education, University of Las Palmas de Gran Canaria, Campus Universitario de Tafira, Las Palmas de Gran Canaria, 35017, Spain; b Research Institute of Biomedical and Health Sciences (IUIBS), University of Las Palmas de Gran Canaria, Paseo Blas Cabrera Felipe “Físico” s/n, 35017, Las Palmas de Gran Canaria, Spain; c Complejo Hospitalario Universitario Insular-Materno Infantil de Las Palmas de Gran Canaria, Clinical Genetics Unit, 35016, Las Palmas de Gran Canaria, Spain; d Department of Physical Performance, The Norwegian School of Sport Sciences, Postboks, 4014 Ulleval Stadion, 0806, Oslo, Norway

**Keywords:** Fatigue, Performance, Ischaemia, AMPK, CaMKII, High-intensity exercise

## Abstract

The Nrf2 transcription factor is induced by reactive oxygen and nitrogen species and is necessary for the adaptive response to exercise in mice. It remains unknown whether Nrf2 signalling is activated by exercise in human skeletal muscle. Here we show that Nrf2 signalling is activated by exercise to exhaustion with similar responses in normoxia (P_I_O_2_: 143 mmHg) and severe acute hypoxia (P_I_O_2_: 73 mmHg). CaMKII and AMPKα phosphorylation were similarly induced in both conditions. Enhanced Nrf2 signalling was achieved by raising Nrf2 total protein and Ser^40^ Nrf2 phosphorylation, accompanied by a reduction of Keap1. Keap1 protein degradation is facilitated by the phosphorylation of p62/SQSTM1 at Ser^349^ by AMPK, which targets Keap1 for autophagic degradation. Consequently, the Nrf2-to-Keap1 ratio was markedly elevated and closely associated with a 2-3-fold increase in Catalase protein. No relationship was observed between Nrf2 signalling and SOD1 and SOD2 protein levels. Application of ischaemia immediately at the end of exercise maintained these changes, which were reverted within 1 min of recovery with free circulation. While SOD2 did not change significantly during either exercise or ischaemia, SOD1 protein expression was marginally downregulated and upregulated during exercise in normoxia and hypoxia, respectively. We conclude that Nrf2/Keap1/Catalase pathway is rapidly regulated during exercise and recovery in human skeletal muscle. Catalase emerges as an essential antioxidant enzyme acutely upregulated during exercise and ischaemia. Post-exercise ischaemia maintains Nrf2 signalling at the level reached at exhaustion and can be used to avoid early post-exercise recovery, which is O_2_-dependent.

## Abbreviations

ADP: Adenosine diphosphate
ATP: Adenosine triphosphate
AMPK: AMP-activated protein kinase
ARE: Antioxidant response element
β-TrCP: β-Transducin repeat-containing protein E3 ubiquitin ligase
BSA: Bovine Serum Albumin
Ca^2+^: Ion calcium
CaMKII: Calmodulin-dependent Protein Kinase II
Cr: Creatine
DEXA: Dual-energy x-ray absorptiometry
F_I_O_2_: Inspired oxygen fraction
GSK3-β: Glycogen synthase kinase-3-β
H_2_O_2_: Hydrogen Peroxide
HR: Heart rate
HRmax: maximal heart rate
HRP: Horseradish peroxidase
Hyp: Hypoxia (P_I_O_2_: 73 mmHg)
IE: Incremental exercise to exhaustion
Keap1: Kelch-like ECH-associated protein 1
mRNA: Messenger RNA
NADH^+^: Nicotinamide adenine dinucleotide reduced
NIRS: near-infrared spectroscopy
Nrf2: Nuclear factor erythroid-derived 2-like 2
Nrf2^−/−^: Nrf2-null mice
Nx: normoxia (P_I_O_2_: 143 mmHg)
p62/SQSTM1: Sequestosome 1
PCr: Phosphocreatine
Pi: Inorganic phosphate
P_I_O_2_: Partial pressure of inspired O_2_
PKCδ: Protein kinase Cδ
PO_2_: Oxygen pressure
PVDF: Polyvinylidene fluoride
RONS: Reactive oxygen and nitrogen species
ROS: Reactive oxygen species
Rpm: Revolutions per minute
SDS: Sodium dodecyl sulfate
SOD1: Superoxide dismutase 1
SOD2: Superoxide dismutase 2
TOI: Tissue oxygenation index
VL: Vastus Lateralis
VO_2_: Oxygen consumption
VO_2_peak: Peak oxygen consumption
W: Watt
Wmax: Maximal power output at exhaustion during the incremental exercise

## 1 Introduction

During exercise reactive oxygen (ROS) and nitrogen species (RNS) (collectively called RONS) are produced depending on the fitness level, the energy substrates oxidized and the characteristics of exercise [[1], [2], [3], [4]]. Although in some circumstances, RONS may cause oxidative damage, RONS also stimulate signalling pathways essential for the adaptive response to exercise [1,5]. One of the main transcription factors involved in RONS-mediated regulation of gene expression is the nuclear factor erythroid-derived 2-like 2 (Nrf2), as shown in Nrf2-null mice (Nrf2^−/−^) [[6], [7], [8]]. In mice skeletal muscle, total Nrf2 protein expression has been reported to increase after 90 min of continuous running [9] and nuclear Nrf2 protein content after 6 h of continuous running [10]. In humans, increased, unchanged and reduced Nrf2 mRNA levels have been reported in skeletal muscle biopsied 3–4 h after exercise [[11], [12], [13], [14]]. However, the changes in Nrf2 protein levels and associated signalling events in response to acute exercise and recovery have not been determined in human skeletal muscle. This is relevant because reduced Nrf2 expression has been associated with lower exercise performance in animal models of chronic disease [15].

The RONS produced during exercise are accompanied by intramuscular changes in oxygen pressure (PO_2_), metabolites and signalling molecules (Ca^2+^, P_i_, Cr, PCr, H^+^, NADH.H^+^, etc.), which return to pre-exercise levels with different time courses [16,17]. Such changes in metabolite accumulation and RONS production are exacerbated when the exercise is performed in hypoxia [18,19], leading to specific adaptations [[20], [21], [22]]. Animal and cell culture experiments indicate that skeletal muscle Nrf2 signalling is upregulated by hypoxia [23,24]. Nevertheless, it remains unknown whether metabolite accumulation and muscle oxygenation influence Nrf2 signalling in response to acute exercise.

Nrf2 signalling is principally regulated by Kelch-like ECH-associated protein 1 (Keap1), which under basal conditions binds to Nrf2 promoting its ubiquitination and proteasomal degradation [25]. Keap1 is a cysteine-rich protein sensitive to modification by electrophiles and oxidants, which cause conformational changes of Keap1 that stabilize the Keap1-Nrf2 interaction, preventing Nrf2 proteasomal degradation. Under lower availability of free Keap1, the newly formed Nrf2 accumulates and translocates to the nucleus where it binds with antioxidant response elements (AREs) to regulate the transcription of more than 250 genes involved in the xenobiotic and antioxidant response, mitochondrial biogenesis, metabolism, detoxification, cytoprotection, inflammation, autophagy, and cell differentiation [25]. Although, it is well established that exercise increases the gene expression of some antioxidant enzymes [11,17,26], the acute effects of exercise on the protein levels of Nrf2/Keap1, and their downstream regulated antioxidant enzymes superoxide dismutase isoenzyme 1 (SOD1), SOD2, and Catalase in human skeletal muscle remain mostly unknown. Moreover, the process of activation/deactivation of Nrf2 signalling in skeletal muscle with contractile activity has not been investigated in humans.

Therefore, the primary purpose of this study was to determine whether Nrf2 is upregulated by acute exercise in human skeletal muscle and the role that Keap1 protein plays in this process. Another aim was to determine whether the level of oxygenation during the exercise influences the Nrf2 signalling response, as well as the role played by muscle oxygenation and metabolite accumulation in the early recovery after exercise.

Given the intrinsic difficulty in assessing RONS production in human skeletal muscle and the low specificity and sensitivity of the oxidative markers at use, we examined potential changes in RONS production by assessing phosphorylation changes known to be mediated by RONS. This is the case of the phosphorylation of Nrf2 at its serine 40 by protein kinase Cδ (PKCδ), a ROS-sensitive kinase [27]. Likewise, we determined the phosphorylation of Ca^2+^/calmodulin-dependent protein kinase II (CaMKII) at its threonine 287. CaMKII is activated by oxidation and autophosphorylation [28], and effect likely amplified by ROS-induced inhibition of phosphatases [29]. During high-intensity exercise, Thr^287^ CaMKII phosphorylation is blunted by the administration of antioxidants before exercise [5]. Likewise, overexpression of antioxidant enzymes prevents Thr^287^ CaMKII phosphorylation in other experimental models [30,31]. As downstream indicators of Nrf2 signalling, we determined the protein expression levels of Catalase, and SOD1 and SOD2. Animal data and cell culture experiments indicate that the gene expression of Catalase [25], SOD1 [32], and SOD2 [33] are stimulated by Nrf2, while the physiological ROS-induced expression of Catalase is blunted in Nrf2^−/−^ mice [6,34]. We also measured Thr^172^ AMPKα phosphorylation as a marker of metabolic stress, since this enzyme is activated principally depending on the AMP/ATP ratio [35], and is necessary to enhance the expression of SOD2 in response to training [36]. Besides, due to the short half-life of RONS and the fast recovery of the energy metabolism upon cessation of exercise, a new experimental model was developed in humans to impede early recovery through the instantaneous application of complete post-exercise ischaemia with a pneumatic cuff in one leg only, using the contralateral leg as a control.

Since Nrf2/Keap1 signalling is expected to be activated by exercise models eliciting redox perturbations [[6], [7], [8]], we used an exercise protocol that allows the achievement of maximal oxygen uptake ($\dot{\text{V}}$O_2_max) in 10–15 min and elicits a marked activation of the glycolysis close to exhaustion [2,37], resulting in oxidative stress [38].

We hypothesised that Nrf2 protein amount and its downstream-regulated proteins Catalase, SOD1, and SOD2 would be increased in response to exhaustive exercise, and more markedly during exercise in hypoxia than normoxia. We also hypothesised that these changes would revert to pre-exercise levels within 1 min of the cessation of exercise in the leg recovering with free circulation. At the same time, Nrf2-depending signalling would increase further in the ischaemic leg due to the additional accumulation of metabolites and the reduction of PO_2_ to anoxic levels.

## 2 Methods

### 2.1 Subjects

Eleven physically active men (means ± SD; age: 21.5 ± 2.0 years, height: 174 ± 8 cm, body mass: 72.3 ± 9.3 kg, body fat: 16.1 ± 4.9%) agreed to participate in this investigation. Subjects were recruited among physical education students with a specific interest in exercise physiology. The inclusion criteria were as follows: a) age between 18 and 35 years, b) sex: male, c) body mass index: < 30 kg cm^−2^, d) normal 12-lead electrocardiogram, and e) having a physically active lifestyle exercising regularly 2–4 times a week, but without following a specific training program at the time of enrolment. The exclusion criteria were: a) smoking, b) any disease or allergy, c) any medical contraindication to exercise, and d) being under medical treatment. All volunteers were informed of the purpose of the study, experimental procedures and potential risks in written and orally before providing their written consent. The study was carried out by the Declaration of Helsinki and was approved by the Ethical Committee of the University of Las Palmas de Gran Canaria.

### 2.2 Study design

The current investigation is part of a larger project initially designed to determine the mechanisms that limit performance during whole-body exercise in humans. The results focusing on exercise performance and muscle metabolism have been already published [[39], [40], [41], [42]]. The present study contains unpublished material and new analyses to determine how Nrf2 signalling is regulated during exercise and recovery in human skeletal muscle. Subjects were requested to avoid physical activity and to refrain from carbonated, caffeinated and alcohol-containing beverages during the 24-h period preceding all experimental days. They were also asked to record their dinner on the day preceding the first experimental day and thoroughly reproduce it before the subsequent experimental days, as well as maintain their usual diet along the experimental phases.

### 2.3 Pre-test and familiarization

On the first visit to the laboratory, anthropometric and body composition assessments were performed by dual-energy X-ray absorptiometry (DEXA) (Hologic QDR-1500, software version 7.10, Hologic Corp., Waltham, MA, USA) [42]. These were continued by familiarization with the exercise tests. The next two sessions were used to determine their peak oxygen consumption ($\dot{\text{V}}$O_2_peak), maximal heart rate (HRmax) and maximal power output reached at exhaustion (Wmax) in normoxia (Nx; F_I_O_2_ = 0.21; P_I_O_2_ ~143 mmHg) and severe acute hypoxia (Hyp; F_I_O_2_ = 0.104; P_I_O_2_ ~73 mmHg). For these purposes, subjects performed ramp incremental exercise tests to exhaustion (IE) on a Lode Excalibur Sport 925900 (Groningen, The Netherlands), as previously reported [42]. In all exercise tests, $\dot{\text{V}}$O_2_ was measured by indirect calorimetry with an open-circuit metabolic cart operated in breath-by-breath mode (Vmax N29; Sensormedics, Yorba Linda, CA, USA). The metabolic cart was calibrated before each test according to the manufacturer's instructions employing high-grade calibration gases using a reference gas mixture made of 16% O_2_ and 4% CO_2_ (Carburos Metálicos, Las Palmas de Gran Canaria, Spain) [42]. The validity of the metabolic cart was established by a butane combustion test [43] and cross-checking end-tidal CO_2_ pressures with arterial blood CO_2_ partial pressures (ABL90, Radiometer, Copenhagen, Denmark) [44]. Respiratory variables were averaged every 20 s for $\dot{\text{V}}$O_2_peak assessment [45].

### 2.4 Main experiments and biopsy sampling

**Fig. 1:**
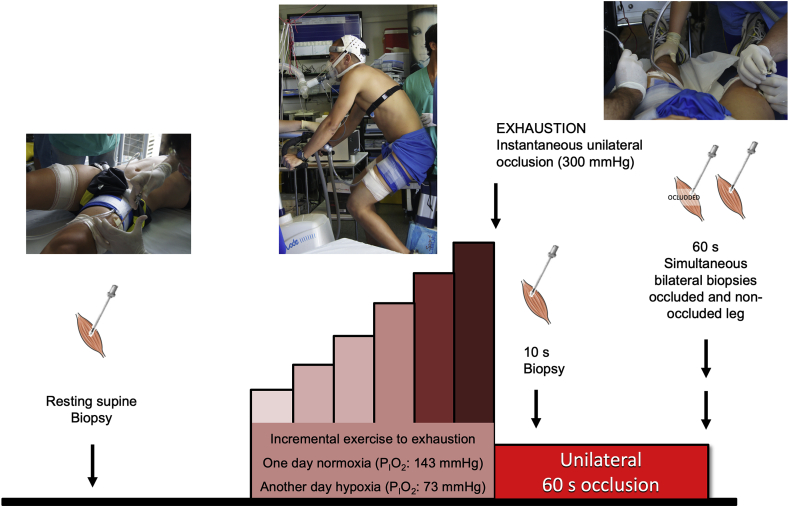
**Schematic representation of the experimental protocol.** Subjects performed an incremental exercise to exhaustion either in normoxia (Nx; F_I_O_2_ = 0.21, P_I_O_2_: 143 mmHg) or severe acute normobaric hypoxia (Hyp; F_I_O_2_ = 0.104, P_I_O_2_: 73 mmHg) in random order. Before warm-up, a resting biopsy was obtained, followed by the exercise test. Immediately at exhaustion, the circulation of one leg was completely occluded by the instantaneous inflation of a cuff at 300 mmHg, which was maintained for 60 s. Skeletal muscle biopsies were taken from the cuffed leg at 10 s and 60 s of occlusion in both trials (Nx and Hyp). In the test performed in hypoxia, another biopsy was obtained 60 s after the end of the incremental exercise from the leg recovering with free circulation, while the subjects recovered breathing room air (i.e., Nx). The application of ischaemia impeded the recovery of muscle metabolites, exhausted the O_2_ stores and resulted in further accumulation of lactate and H^+^, Pi, and Cr.

The muscle biopsies analysed here were obtained in two main experimental sessions administered in random order and separate days (Fig. 1). Each session included one incremental exercise to exhaustion on an isokinetic ergometer (Lode Excalibur Sport 925900, Groningen, The Netherlands), one day in normoxia (Nx; F_I_O_2_ = 0.21; barometric pressure 735–745 mmHg) and the other in hypoxia (Hyp; F_I_O_2_ = 0.104; barometric pressure 735–745 mmHg). Exhaustion was defined by the subject stopping pedalling or dropping pedalling rate below 50 rpm despite strong verbal encouragement for 5 s.

On each experimental day, subjects reported to the laboratory at 08.00 h, after overnight fasting from 22.00 h. In the Nx session, after 10 min of rest in the supine position, the skin of the lateral aspect of both thighs (middle third) was anaesthetized (lidocaine (lignocaine) 2%, 2 ml, without adrenaline), avoiding infiltrating the muscle belly with lidocaine. Fifteen minutes later, a 5-mm incision of the skin and superficial fascia was performed, and the Bergstrom's type biopsy needle inserted to obtain a muscle biopsy from the m. *vastus lateralis* of one of the two thighs, which were randomly assigned (Bergstrom technique with suction). This biopsy was labelled as Pre Nx. For this first biopsy, the needle was directed distally with 45° of inclination. An additional incision was similarly performed in the contralateral leg before the start of the exercise. Both incisions were covered with temporary plasters. Then, a cuff (SCD10, Hokanson, Bellevue, WA, USA) connected to a rapid cuff inflator (Hokanson, E20 AG101) was placed unilaterally around the leg biopsied first and taped as close as possible to the inguinal crease. Subsequently, the volunteers seated on the cycle ergometer, which was pre-configured with saddle height and handlebar position used in familiarization tests. Then, after verification of electrocardiograph and metabolic cart connections, the resting measurements started, and after 2-min of data collection, the incremental exercise in normoxia begun with an initial load of 80 W, which was increased by 30 W every 2 min until exhaustion. At exhaustion, the cuff was inflated instantaneously at 300 mmHg, and a countdown started to obtain a second biopsy (labelled as Post Nx) exactly 10 s after exhaustion, i.e. after 10 s of ischaemia. For the second biopsy (Post Nx), the Bergstrom-type needle was introduced perpendicular to the thigh. Subsequently, while maintaining the cuff inflated the subject rested quietly on the cycle ergometer and the third biopsy was taken exactly 60 s after exhaustion directing the biopsy needle proximally (45° inclination) (labelled as Oc1m Nx) [46]. This novel experimental approach permitted the assessment of muscle signalling changes during ischaemia, while the energy metabolism relied only on anaerobic sources, i.e., the energy supplied by phosphagens (ATP and phosphocreatine (PCr)) and the glycolysis [42].

In the hypoxia session, similar procedures were applied for subject preparation and biopsy sampling at rest, the latter obtained while the volunteers were breathing normoxic air (labelled as Pre Hyp). The hypoxic test started with 2 min collection at rest (P_I_O_2_ ~73 mmHg, AltiTrainer200, SMTEC, Nyon, Switzerland), followed by 2 min at 60 W, and after that, increments of 20 W every 2 min until exhaustion. At exhaustion, the cuff was instantaneously inflated, and the second biopsy was taken at the 10^th^ s (labelled as Post Hyp). Then, while maintaining the cuff inflated the subjects were carefully moved to a stretcher prepared beside the cycle ergometer where the third biopsy was obtained exactly after 60 s of ischaemia (labelled as Oc1m Hyp). Sixty seconds after exhaustion, a fourth biopsy was obtained from the contralateral leg, which had been recovering in normoxia and without occlusion (labelled as FC1m). This means that the 3^rd^ and 4^th^ biopsies were taken simultaneously. Upon collection, all muscle samples (80–160 mg) were immediately dried on a sterile gauze, carefully freed from visible blood, dissected of any debris and fat tissue and immediately frozen in liquid nitrogen and stored at −80 °C until further analysis.

### 2.5 Muscle metabolites, protein extraction and western blotting

Determination of muscle metabolites was performed as previously described [42]. Extracts of muscle protein (whole skeletal muscle lysates) were prepared as reported elsewhere [5], and total protein content quantified using the bicinchoninic acid assay [47]. In brief, ~10 mg of muscle were ground by stainless steel balls during 1 min in a Mikro-Dismembrator S (Sartorius, Goettingen, Germany) and immediately homogenised in urea lysis buffer (6 M urea, 1% SDS) and 50X Complete protease inhibitor (Cat. #4906837001) and 10X PhosSTOP phosphatase inhibitor (Cat. #4906837001) cocktails (Roche, Basel, Switzerland). Almost equal final concentration in all muscle protein extracts was obtained (4.01 μg/uL) by following an individual adjustment of the extract volume using a volume calibration curve. Then, the lysate was centrifuged for 12 min at 25,200 g at 16 °C. The resulting supernatant was diluted with electrophoresis loading buffer (160 mM Tris-HCl, pH 6.8, 5.9% SDS, 25.5% glycerol, 15% β-mercaptoethanol-bromophenol blue).

For Western Blotting, the optimal amount of total protein to be loaded and the antibody concentration for each assay was determined by loading protein extracts in different amounts ranging from 2 to 45 μg. After verification of linearity within this range, equal amounts of protein of each sample (10–25 μg) were electrophoresed on SDS-PAGE gels using the system of Laemmli and transferred to Immun-Blot polyvinylidene fluoride (PVDF) membranes for protein blotting (Bio-Rad Laboratories, Hercules, CA, USA) (a more detailed description of procedures is available in the Supplementary Table 1). Control samples (whole skeletal muscle lysates from healthy young men prepared as the experimental samples) and a total protein staining-technique method (Reactive Brown 10, Sigma Aldrich, St. Louis, MO, USA) were used to accurately quantify the variability of the assays and ensure optimal loading and transfer efficiency. For protein expression determination, the samples from each subject were run together onto the same gel with control samples in quadruplicate.

Membranes were blocked for 1 h in 4% bovine serum albumin or 5% blotting-grade blocker diluted in Tris-buffered saline containing 0.1% Tween 20 (TBS-T) (BSA-or Blotto-blocking buffer) and incubated overnight for 12–15 h at 4 °C with primary antibodies. Antibodies were diluted in 4% BSA-blocking buffer or 5% Blotto-blocking buffer. After incubation with primary antibodies, the membranes were incubated with an HRP-conjugated anti-rabbit or anti-mouse antibody (diluted 1:5000 in 5% Blotto blocking buffer in all instances) and subsequent chemiluminescent visualization using Clarity™ Western ECL Substrate (Bio-Rad Laboratories, Hemel Hempstead, Hertfordshire, UK) using a ChemiDoc™ Touch Imaging System (Bio-Rad Laboratories, Hercules, CA, USA). Finally, band densitometric data were quantified in an exposition prior to saturation of the signal with the Image Lab © software 6.0.1 (Bio-Rad Laboratories, Hercules, CA, USA) as arbitrary units (a.u). Since loading was homogeneous in all membranes, no further corrections were performed. In the case of Thr^172^ AMPKα phosphorylation, the antibody was stripped off using a buffer containing Tris-HCL 1 M pH 6.7, SDS 20%, β-mercaptoethanol 14.3 M and H_2_O. Subsequently, membranes were stained with Reactive Brown, re-blocked and re-incubated with HRP conjugated antibody to check for stripping efficiency and subsequently re-blocked and re-incubated with the corresponding antibody for total AMPKα.

### 2.6 Materials

The Protein Plus Precision All Blue Standards were acquired from Bio-Rad Laboratories (Hemel Hempstead Hertfordshire, UK). The antibodies employed in this investigation were obtained from different manufacturers. The corresponding catalogue numbers from Abcam (Cambridge, USA) were as follows: pSer^40^ Nrf2 (no. ab76026), Nrf2 (no. ab62352), Keap1 (no. ab119403), SOD1 (no. ab16831), Sequestosome 1 (SQSTM1/p62) (no. ab56416) and SQSTM1/p62 (pSer^349^) (no. ab211324). The antibodies purchased from Cell Signaling Technology (Danvers, MA, USA) were: Thr^287^ CaMKII (no. 12716), AMPKα (no. 2532), Thr^172^ AMPKα (no. 2535), Catalase (no.14097) and SOD2 (no. 13141). Some secondary HRP-conjugated goat anti-rabbit (no. 111-035-144) and the HRP-conjugated goat anti-mouse (no.115-035-003) antibodies were acquired from Jackson ImmunoResearch (West Grove, PA, USA). Other secondary HRP-conjugated goat antibodies were obtained from Santa Cruz Biotechnology (Dallas, TX, USA): anti-rabbit (no. sc2004) and anti-mouse (no. sc2031). See Supplementary Table 1 for a more detailed description of the antibodies and procedures.

### 2.7 Statistical analysis

Variables were checked for Gaussian distribution using the Shapiro–Wilks test, and when appropriate, data were transformed logarithmically before further analysis. A two-way 3 x 2 repeated-measures ANOVA with time (Pre, Post, and Oc1m) and F_I_O_2_ (Normoxia and hypoxia) as within-subject factors was applied to examine the main effects and interactions. Additionally, the same type of ANOVA was also conducted with two levels for time, to study the effect of exercise on the signalling responses (using the average of Post and Oc1m conditions). In the case of SOD1 and SOD2, the data were normalized to the mean pre-exercise value of each condition and expressed as fold changes. The normalized values for these two measurements were then tested with the same type of repeated-measures ANOVA. The Mauchly's test of sphericity was run before the ANOVAs. In the case of violation of the sphericity assumption, the degrees of freedom were adjusted according to the Huynh and Feldt test. When a significant main or interaction effects were detected, pairwise comparisons at specific time points were adjusted for multiple comparisons using the Holm-Bonferroni procedure. The two biopsies obtained 1 min after ischaemia were compared with paired Student's *t*-test. Pearson's correlation analysis was applied to check for linear associations between variables. Values are reported as the mean ± standard deviation (SD) unless otherwise stated. Statistical significance was set at p < 0.05. All statistical analyses were performed using IBM SPSS Statistics v.21 for Mac (SPSS Inc., Chicago, IL, USA).

## 4 Results

### 4.1 Muscle metabolites

The responses regarding the metabolite accumulation in both conditions have been reported previously [42] and will only be summarized here. Briefly, following IE (i.e. at Post), muscle lactate, phosphocreatine (PCr) and ATP changed similarly in Nx and Hyp. From Post to the subsequent biopsy (ischaemic or free circulation period), muscle lactate increased only at Oc1m (25%; p < 0.05). PCr was reduced by 94 and 48%, compared to Pre levels, in Oc1m and FC1m, respectively (p < 0.005), regardless of exercise F_I_O_2,_ indicating recovery to 47.7% of the Pre-exercise PCr levels in the leg recovering with free circulation. Femoral vein PO_2_ was 21.1 ± 2.0 and 10.6 ± 2.8 mmHg at Wmax, in Nx and Hyp, respectively (p < 0.001).

### 4.2 Muscle signalling

#### 4.2.1 CaMKII, AMPK and p62

**Fig. 2:**
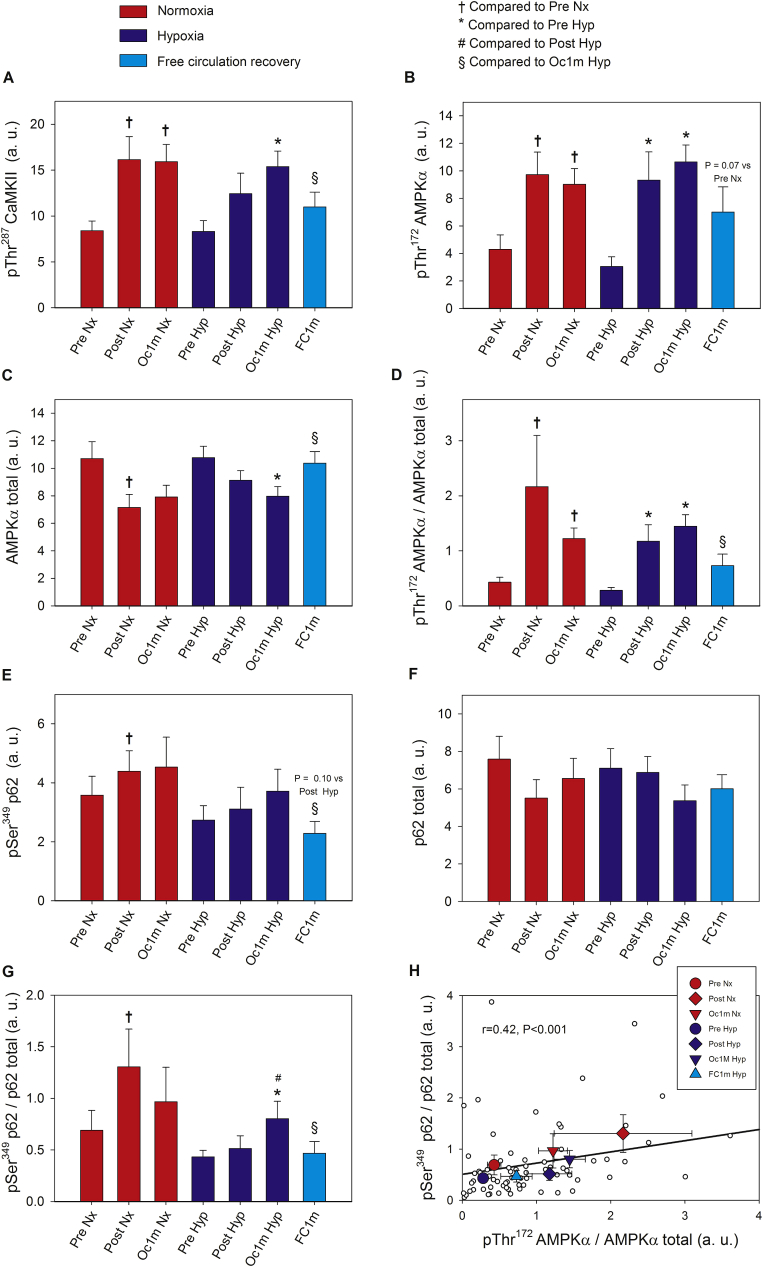
**Skeletal muscle intracellular CaMKII, AMPKα and p62 signalling in response to incremental exercise to exhaustion in normoxia and severe hypoxia and the application of immediate ischaemic or non-ischaemic recovery.** Protein expression levels of pThr^287^ CaMKII **(A)**, pThr^172^ AMPKα **(B)**, AMPKα total **(C)**, pThr^172^ AMPKα/AMPKα total ratio **(D)**, pSer^349^ p62 **(E)**, p62 total (**F**), pSer^349^ p62/p62 total ratio (**G**), and association between the ratio of pThr^172^ AMPKα/AMPKα total and the ratio of pSer^349^ p62/p62 total **(H)**. Nx; test performed in normoxia (F_I_O_2_ = 0.21, P_I_O_2_: 143 mmHg), Hyp; test performed in severe acute normobaric hypoxia (F_I_O_2_ = 0.104, P_I_O_2_: 73 mmHg); Pre, before exercise; Post, 10 s after the end of exercise with ischaemic recovery; Oc1m, 60 s after the end of exercise with ischaemic recovery; FC1m, 60 s after the end of exercise in the leg recovering without occlusion (free circulation); p62/SQSTM1 (shortened to p62). n = 11 in all conditions except for Oc1m Nx (n = 9), Post Hyp (n = 10), and Oc1m Hyp (n = 10). In (**H**), large symbols represent the mean of the subjects studied in each condition. The correlation coefficient and regression line have been calculated using the individual values (small white circles, n = 73). A detailed description of the experimental phases is explained in Fig. 1. The statistical analysis was performed with logarithmically transformed data for the pThr^172^ AMPKα/AMPKα total ratio and p62 total. The values shown are means ± standard errors and expressed in arbitrary units (a.u.). †p < 0.05 vs Pre Nx; *p < 0.05 vs Pre Hyp; ^#^p < 0.05 vs Post Hyp; ^§^p < 0.05 vs Oc1m Hyp.

pThr^287^ CaMKII expression was increased by 1.7-fold after IE, remaining at this level after 1 min of occlusion (1.9-fold above Pre), with a similar response in Nx and Hyp (ANOVA F_I_O_2_ effect p = 0.83, time effect p = 0.001, F_I_O_2_ by time interaction p = 0.9) (Fig. 2A). Solely in the leg recovering with free circulation, pThr^287^ CaMKII levels returned to pre-exercise values 1 min after the end of the IE.

Compared to Pre, pThr^172^ AMPKα expression was increased by 2.4 and 3.0-fold at Post and Oc1m (Post vs Oc1m p = 0.07; ANOVA time effect p < 0.001), with a similar response in Nx and Hyp (ANOVA F_I_O_2_ effect p = 0.71, F_I_O_2_ by time interaction p = 0.68, Fig. 2B). After the IE performed in Hyp, no significant differences were observed between the occluded and non-occluded leg 1 min after exercise (p = 0.19) (Fig. 2B).

Compared to Pre, AMPKα total expression was reduced by 25 and 27% at Post and Oc1m (ANOVA time effect p = 0.007), with a similar response in Nx and Hyp (F_I_O_2_ effect p = 1.0, ANOVA F_I_O_2_ by time interaction p = 0.72, Fig. 2C). One minute after the end of the IE, AMPKα total expression returned to the pre-exercise levels only in the non-occluded leg (Fig. 2C).

The ratio pThr^172^ AMPKα/AMPKα total was increased by 4.5 and 4.0-fold at Post and Oc1m (Post vs Oc1m p = 0.38; ANOVA time effect p < 0.004), with a similar response in Nx and Hyp (ANOVA F_I_O_2_ effect p = 0.71, F_I_O_2_ by time interaction p = 0.42, Fig. 2D). After the IE performed in Hyp, the pThr^172^ AMPKα/AMPKα total ratio tended to be 52% lower in the leg recovering without occlusion (p = 0.057) (Fig. 2D).

The protein expression of pSer^349^ p62 showed a tendency to increase with time (ANOVA time effect p = 0.051). When the mean of the two Pre conditions was compared to the mean of Post and Oc1m conditions, pSer^349^ p62 protein was 26% higher after exercise (p = 0.04) (Fig. 2E). Following the IE performed in Hyp, pSer^349^ p62 protein was 41% lower in the leg recovering without occlusion compared with the occluded leg (p = 0.026), and similar to that observed at Pre (Fig. 2E). No significant changes in p62 were detected using raw data (ANOVA F_I_O_2_ effect p = 0.84, time effect p = 0.07, F_I_O_2_ by time interaction p = 0.50). A secondary analysis was carried out to compare the mean of the two Pre conditions with the mean of the two post-exercise conditions (Post and Oc1m), which showed that p62 was reduced by ~20% after exercise (p = 0.02) (Fig. 2F). The ratio pSer^349^ p62/p62 total was increased by 1.8 and 1.5-fold at Post and Oc1m (Post vs Oc1m p = 0.17; ANOVA time effect p = 0.029), with a similar response in Nx and Hyp (F_I_O_2_ effect p = 0.34, ANOVA F_I_O_2_ by time, p = 0.19, Fig. 2G). Following the IE performed in Hyp, the ratio pSer^349^ p62/p62 total was 43% lower in the leg recovering without occlusion (p = 0.017), and similar to that observed before the exercise (Fig. 2G). After the IE performed in Hyp, the pSer^349^ p62/p62 total ratio increased by 1.5-fold during the period of ischaemia (p = 0.026). There was an association between the ratio pThr^172^ AMPKα/AMPKα total and the ratio pSer^349^ p62/p62 total across conditions (r = 0.42, p < 0.001, n = 73) (Fig. 2H).

#### 4.2.2 Antioxidant enzymes: Catalase, SOD1 and SOD2

**Fig. 3:**
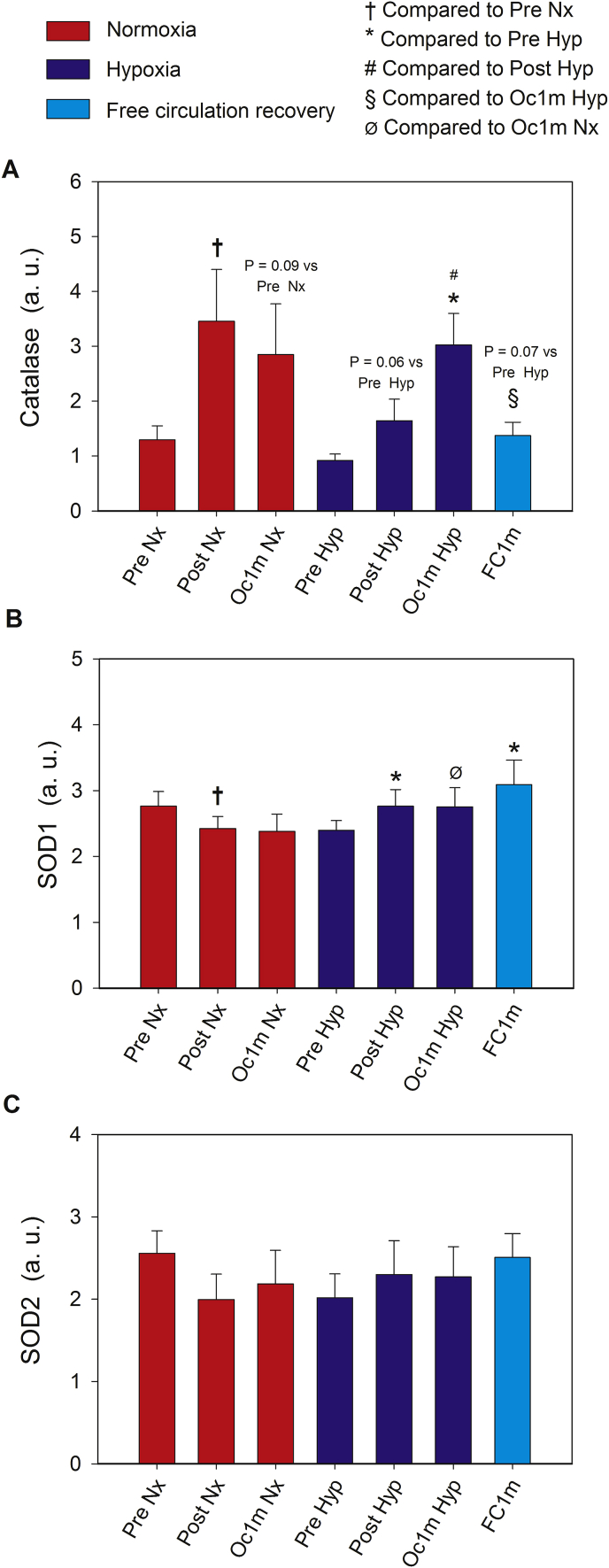
**Skeletal muscle intracellular Catalase, SOD1 and SOD2 signalling in response to incremental exercise to exhaustion in normoxia and severe hypoxia and the application of immediate ischaemic or non-ischaemic recovery.** Protein expression levels of Catalase **(A)**, SOD1 **(B)**, and SOD2 **(C)**. Nx; test performed in normoxia (F_I_O_2_ = 0.21, P_I_O_2_: 143 mmHg), Hyp; test performed in severe acute normobaric hypoxia (F_I_O_2_ = 0.104, P_I_O_2_: 73 mmHg); Pre, before exercise; Post, 10 s after the end of exercise with ischaemic recovery; Oc1m, 60 s after the end of exercise with ischaemic recovery; FC1m, 60 s after the end of exercise in the leg recovering without occlusion (free circulation). A detailed description of the experimental phases is explained in Fig. 1. The statistical analysis was performed with logarithmically transformed data for Catalase and SOD2. The values shown are means ± standard errors and expressed in arbitrary units (a.u.). n = 11 in all conditions except for Oc1m Nx (n = 9), Post Hyp (n = 10), and Oc1m Hyp (n = 10). †p < 0.05 vs Pre Nx; *p < 0.05 vs Pre Hyp; ^#^p < 0.05 vs Post Hyp; ^§^p < 0.05 vs Oc1m Hyp; ^Ø^ p < 0.05 vs Oc1m Nx.

Catalase protein expression was increased by 2.3 and 2.8-fold immediately after IE (Post) and after 1 min of ischaemic recovery (Oc1m), respectively, with a similar response in Nx and Hyp (ANOVA time effect p = 0.001, F_I_O_2_ by time interaction p = 0.12). Since the level of Catalase tended to be lower in the Pre value obtained the day of the experiment in Hyp (p = 0.10), we additionally analysed the experiment performed in Hyp separately. During the IE in Hyp, compared to Pre, Catalase expression levels were elevated 1.9-fold and 3.3-fold immediately after the IE (Post) and after 1 min of occlusion (Oc1m), respectively. From the end of the IE in Hyp, the level of Catalase doubled during the occlusion (Post vs Oc1m, p = 0.018) (Fig. 3A). One minute after the IE in Hyp, Catalase protein levels were reduced by 56% in the non-occluded leg (FC1m), remaining 1.5-fold above Pre levels (p = 0.048) (Fig. 3A).

Although no significant changes were observed in SOD1 protein levels with exercise nor ischaemia, SOD1 tended to decrease and increase after the IE in Nx and Hyp, respectively (ANOVA F_I_O_2_ effect p = 0.58; time effect p = 0.86, F_I_O_2_ by time interaction p = 0.053). To reduce variability between starting values on the testing day, we also repeated the analysis for fold changes regarding the pre-exercise value of each day. This analysis showed that SOD1 was reduced and increased after the IE in Nx and Hyp, respectively (ANOVA F_I_O_2_ effect p = 0.002, time effect p = 0.90, F_I_O_2_ by time interaction p = 0.044). The mean values of the Post and Oc1m after the IE in Nx and Hyp were compared using a paired Student's *t*-test. This analysis found a 30% higher SOD1 protein content after exercise + occlusion in Hyp than in Nx (p = 0.001). In both experiments, SOD1 remained unchanged from 10 to 60 s of ischaemia. One min after IE in Hyp, no significant differences were observed in SOD1 expression between the occluded and the non-occluded leg (p = 0.11) (Fig. 3B). No significant changes were observed in SOD2 protein levels with exercise nor ischaemia (ANOVA F_I_O_2_ effect p = 0.34; time effect p = 0.45, F_I_O_2_ by time interaction p = 0.22) (Fig. 3C). The protein expressions of SOD1 and SOD2 were positively associated (r = 0.51, p < 0.001, n = 73).

#### 4.2.3 Nrf2/Keap1 signalling

**Fig. 4:**
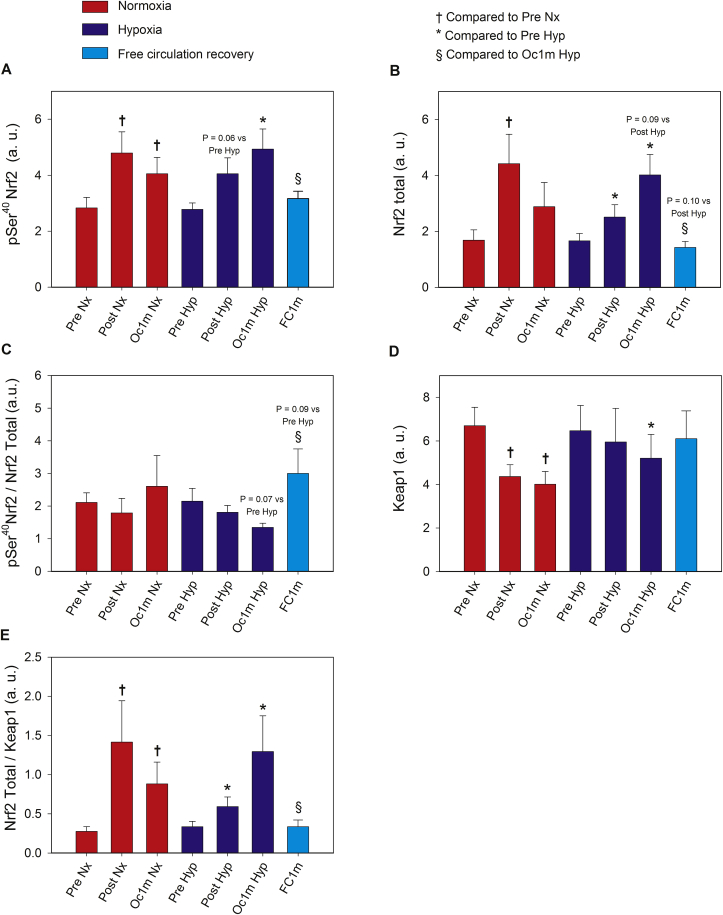
**Skeletal muscle intracellular Nrf2 and Keap1 signalling in response to incremental exercise to exhaustion in normoxia and severe hypoxia and the application of immediate ischaemic or non-ischaemic recovery.** Protein expression levels of pSer^40^ Nrf2 **(A)**, Nrf2 total **(B),** pSer^40^ Nrf2/Nrf2 total ratio (**C)**, Keap1 **(D),** Nrf2 Total/Keap1 ratio (**E**). Nx; test performed in normoxia (F_I_O_2_ = 0.21, P_I_O_2_: 143 mmHg), Hyp; test performed in severe acute normobaric hypoxia (F_I_O_2_ = 0.104, P_I_O_2_: 73 mmHg); Pre, before exercise; Post, 10 s after the end of exercise with ischaemic recovery; Oc1m, 60 s after the end of exercise with ischaemic recovery; FC1m, 60 s after the end of exercise in the leg recovering without occlusion (free circulation). A detailed description of the experimental phases is explained in Fig. 1. The statistical analysis was performed with logarithmically transformed data for all proteins except for Keap1. The values shown are means ± standard errors and expressed in arbitrary units (a.u.). n = 11 in all conditions except for Oc1m Nx (n = 9), Post Hyp (n = 10), and Oc1m Hyp (n = 10). †p < 0.05 vs Pre Nx; *p < 0.05 vs Pre Hyp; ^#^p < 0.05 vs Post Hyp; ^§^p < 0.05 vs Oc1m Hyp.

The levels of phosphorylated Nrf2 at Ser^40^ were increased in Post and Oc1m compared to Pre by 1.5 and 1.6-fold, respectively, with a similar response in Nx and Hyp (ANOVA F_I_O_2_ by time, p = 0.7; ANOVA time effects p < 0.01, Fig. 4A). The exercise-elicited increase of pSer^40^ Nrf2 was maintained at the same level after 1 min of ischaemia, while it recovered to pre-exercise values in the non-occluded leg. Similar changes were observed in the expression of Nrf2 total protein (Fig. 4B). Although no statistically significant changes were observed in the pSer^40^ Nrf2/Nrf2 total ratio between Nx and Hyp, 1 min after IE in Hyp, this ratio was higher in the occluded compared to the non-occluded leg (p = 0.02) (Fig. 4C).

Compared to Pre, Keap1 expression was diminished by 23 and 29% at Post and Oc1m, respectively (ANOVA time effects p = 0.015; ANOVA F_I_O_2_ by time interaction p = 0.52). One minute after exercise, Keap1 recovered pre-exercise values in the non-occluded leg (Fig. 4D).

The Nrf2 total protein/Keap1 ratio was augmented by 3.3-fold (p = 0.02) at Post, remaining at this level (3.4-fold above Pre) after 1 min of occlusion. This response was similar for the exercise performed in Nx and Hyp (ANOVA time effects p = 0.002; F_I_O_2_ by time interaction p = 0.45). One minute after the end of the IE, Nrf2 total protein/Keap1 ratio returned to the pre-exercise levels in the non-occluded leg (Fig. 4E).

#### 4.2.4 Phosphorylated CaMKII at Thr^287^ is closely associated with Nrf2 and Keap1 proteins

**Fig. 5:**
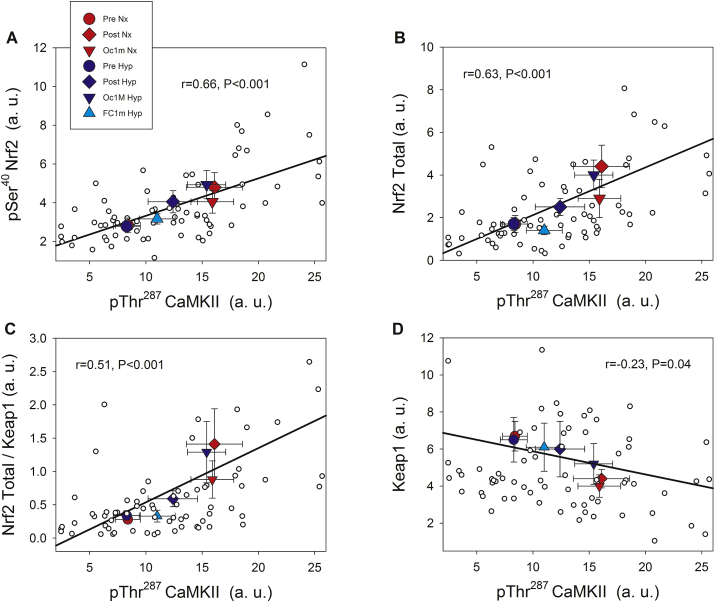
**Associations between the protein expression levels of phosphorylated CaMKII, Nrf2 and Keap1 across experimental phases.** pThr^287^ CaMKII and pSer^40^ Nrf2 **(A)** pThr^287^ CaMKII and Nrf2 total **(B)**, pThr^287^ CaMKII and Nrf2/Keap1 ratio **(C)**, pThr^287^ CaMKII and Keap1 **(D)**. A description of the experimental phases is explained in Fig. 1. Nx; test performed in normoxia (F_I_O_2_ = 0.21, P_I_O_2_: 143 mmHg), Hyp; test performed in severe acute normobaric hypoxia (F_I_O_2_ = 0.104, P_I_O_2_: 73 mmHg); Pre, before exercise; Post, 10 s after the end of exercise with ischaemic recovery; Oc1m, 60 s after the end of exercise with ischaemic recovery; FC1m, 60 s after the end of exercise in the leg recovering without occlusion (free circulation). n = 11 in all conditions except for Oc1m Nx (n = 9), Post Hyp (n = 10), and Oc1m Hyp (n = 10). Large symbols: each point is representing the mean of the subjects studied in each condition. Correlation coefficients and regression lines have been calculated using the individual values (small white circles, n = 73). The values shown are means ± standard errors and expressed in arbitrary units (a.u.). Statistical significance was set at p < 0.05.

As illustrated in Fig. 5, pThr^287^ CaMKII protein levels were positively associated with those of pSer^40^ Nrf2 expression (r = 0.66, p < 0.001, n = 73), Nrf2 total protein (r = 0.63, p < 0.001, n = 73), and the Nrf2 total protein/Keap1 ratio (r = 0.51, p < 0.005, n = 73) and negatively with Keap1 (r = −0.23, p = 0.04) across conditions.

#### 4.2.5 Nrf2 signalling during exercise is closely associated with Catalase protein expression but not with SOD1 or SOD2 protein levels

**Fig. 6:**
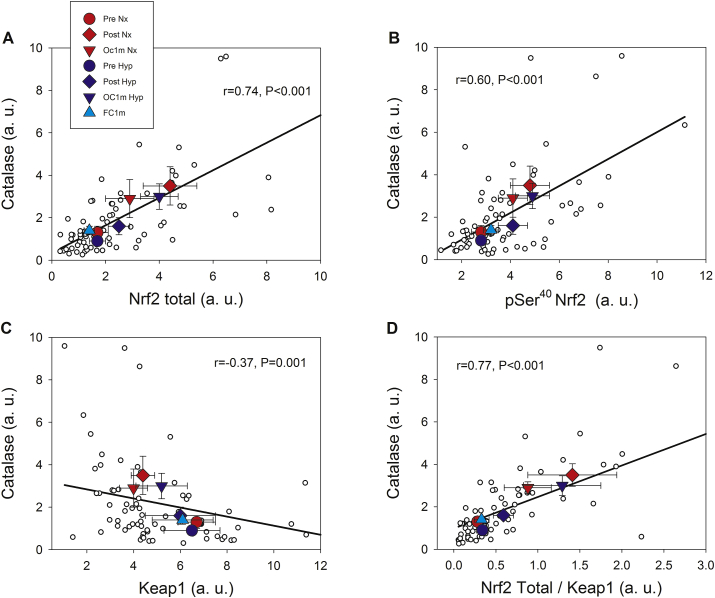
**Associations between the protein expression levels of Catalase, Nrf2 and Keap1 across experimental phases.** Nrf2 total and Catalase **(A),** pSer^40^ Nrf2 and Catalase **(B)**, Keap1 and Catalase **(C)** and Nrf2/Keap1 ratio and Catalase (**D**). A description of the experimental phases is explained in Fig. 1. Nx; test performed in normoxia (F_I_O_2_ = 0.21, P_I_O_2_: 143 mmHg), Hyp; test performed in severe acute normobaric hypoxia (F_I_O_2_ = 0.104, P_I_O_2_: 73 mmHg); Pre, before exercise; Post, 10 s after the end of exercise with ischaemic recovery; Oc1m, 60 s after the end of exercise with ischaemic recovery; FC1m, 60 s after the end of exercise without ischaemic recovery (free circulation). n = 11 in all conditions except for Oc1m Nx (n = 9), Post Hyp (n = 10), and Oc1m Hyp (n = 10). Large symbols: each point is representing the mean of the subjects studied in each condition. Correlation coefficients and regression lines have been calculated using the individual values (small white circles, n = 73). The values shown are means ± standard errors and expressed in arbitrary units (a.u.). Statistical significance was set at p < 0.05.

**Fig. 7:**
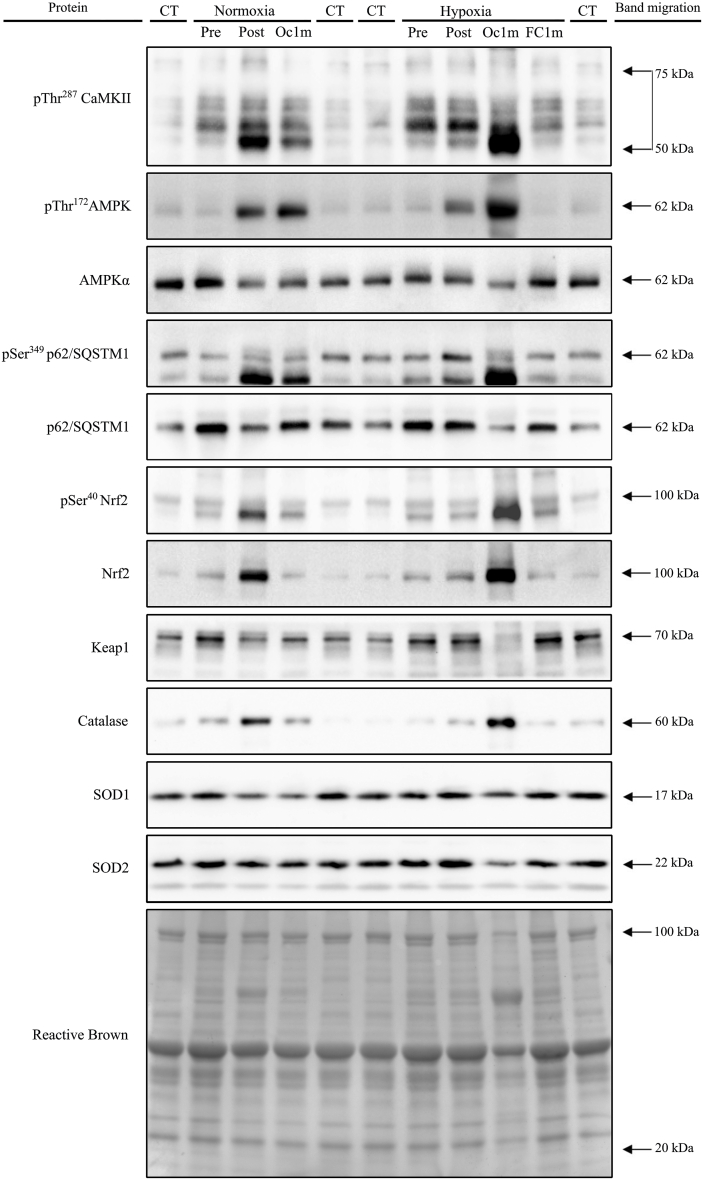
**Representative immunoblot images of all proteins and their regulatory phosphorylations and the total amount of protein loaded (Reactive Brown staining) from a single participant.** From top to bottom: pThr^287^ CaMKII, pThr^172^ AMPKα, AMPKα total, pSer^349^ p62/SQSTM1, p62/SQSTM1 total, pSer^40^ Nrf2, Nrf2, Keap1, Catalase, SOD1, SOD2 and Reactive Brown (as protein loading control). Nx; test performed in normoxia (F_I_O_2_ = 0.21, P_I_O_2_: 143 mmHg), Hyp; test performed in severe acute normobaric hypoxia (F_I_O_2_ = 0.104, P_I_O_2_: 73 mmHg); Pre, before exercise; Post, 10 s after the end of exercise with ischaemic recovery; Oc1m, 60 s after the end of exercise with ischaemic recovery; FC1m, 60 s after the end of exercise without ischaemic recovery (free circulation); CT, control sample. Arrows indicate estimated molecular weights.

Catalase protein expression was closely associated with Nrf2 total protein expression (r = 0.74, p < 0.001, n = 73), pSer^40^ Nrf2 (r = 0.60, p < 0.001, n = 73), Keap1 (r = −0.37, p = 0.001, n = 73), and the Nrf2 total protein/Keap1 ratio (r = 0.77, p < 0.001, n = 73) (Fig. 6). No associations were observed between Nrf2 total protein and SOD1 (r = −0.001, p = 0.99, n = 73); pSer^40^ Nrf2 protein and SOD1 (r = −0.15, p = 0.20, n = 73), Nrf2 total protein/Keap1 ratio and SOD1 (r = −0.08, p = 0.47, n = 73), and between pThr^287^ CaMKII and SOD1 (r = −0.10, p = 0.39, n = 73). No associations were observed between pSer^40^ Nrf2 protein and SOD2 (r = −0.18, p = 0.13), while there was a negative association between SOD2 and Nrf2 total protein (r = −0.25, p = 0.03, n = 73), Nrf2 total protein/Keap1 ratio and SOD2 (r = −0.33, p = 0.004, n = 73), and pThr^287^ CaMKII and SOD2 (r = −0.18, p = 0.12, n = 73). Representative immunoblots of all proteins studied are presented in Fig. 7.

## 5 Discussion

**Fig. 8:**
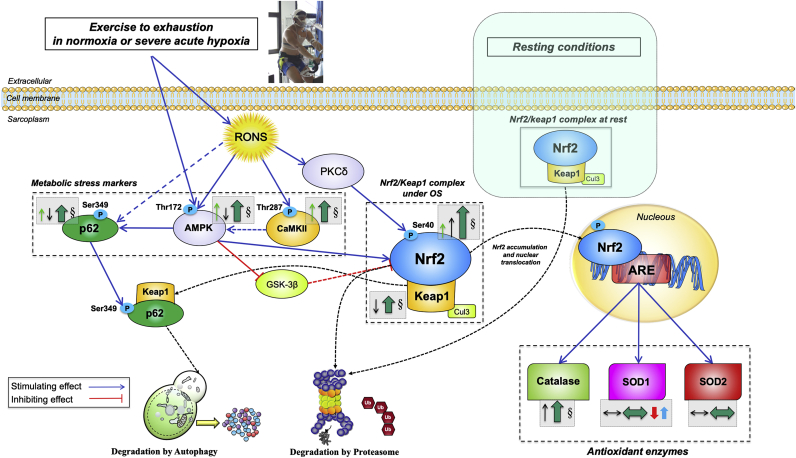
**Schematic model of the regulation of Nrf2 and Keap1 signalling in human skeletal muscle immediately after incremental exercise to exhaustion in normoxia and severe hypoxia.** Under basal conditions, Keap1 continuously targets Nrf2 for ubiquitination and degradation, allowing for minimal levels of Nrf2. The production of RONS during exhaustive exercise stimulates the activation of AMPK and CaMKII. Concomitantly, CaMKII acts indirectly as an upstream AMPK activator (by an unknown mechanism). AMPK promotes the increase of Nrf2 levels by two main mechanisms. Firstly, by phosphorylating p62 at Ser^349^, which stimulates the p62-mediated degradation of Keap1 via autophagy; secondly, by phosphorylating and blocking GSK3-β, which activates β-TrCP (an E3 ubiquitin-protein ligase) which tags Nrf2 for proteasomal degradation (not measured here). RONS may also activate PKCδ which phosphorylates Nrf2 at its Serine 40 promoting its nuclear translocation and genes transactivation. The lowered levels of Keap1 and the reduced amount of p62 observed here are suggestive of co-degradation following exercise. Overall, the augmented Nrf2 total and phosphorylated protein expression together with the rise in the Nrf2-to-Keap1 ratio elicited by exhausting exercise should be sufficient to enhance the Nrf2-mediated antioxidant response. A central role of Catalase is manifested by a remarkable increase in its protein content following exercise, which was exacerbated during exercise in severe acute hypoxia, likely as a response to increased H_2_O_2_ production, by superoxide dismutases. This process is facilitated in hypoxia due to the upregulation of SOD1. No acute changes in SOD2 protein expression were observed. Most changes evoked by the exhaustive exercise bout were almost entirely reverted to baseline in less than 60 s by an O_2_-dependent mechanism. Activating/inhibiting actions are represented by blue/red connecting lines (dashed if the effect is indirect). Changes on cellular locations are presented with black dashed lines. The arrows and symbols depicted inside dashed grey boxes and located beside the specific markers illustrate the overall protein expression changes in this investigation, as follows: Thin arrows in green (phosphorylated form) and black (total form) depict the overall direction of the outcomes (increase/decrease) for the particular muscle protein; thick arrows in darker green represent the overall effect on stimulation/inhibition of Nrf2 signalling; the symbol § indicates a significant difference between the biopsies taken 60 s after the end of the exercise, i.e., between the legs recovering with and without ischaemia. A differential modulation due to F_I_O_2_ is illustrated by the presence of arrows in red (normoxia) and blue (severe hypoxia). The size of each arrow is commensurate with the magnitude of the change. Abbreviations not defined in the text: ARE, antioxidant response element; OS: oxidative stress. (For interpretation of the references to colour in this figure legend, the reader is referred to the Web version of this article.)

This study shows that Nrf2 signalling is activated by exercise to exhaustion in human skeletal muscle. To our knowledge, this is the first study examining the response of the Nrf2 signalling to intense acute exercise in human skeletal muscle and its relationship with metabolite accumulation, O_2_ delivery and tissue O_2_ pressures. In contrast to our hypothesis, the degree of activation of Nrf2 signalling was essentially similar in normoxia and hypoxia, despite a 50% lower femoral vein PO_2_ during the exercise in severe acute hypoxia. Increased Nrf2 signalling was achieved by enhancing the total Nrf2 protein content while reducing that of Keap1, the main inhibitor of Nrf2 signalling. The combination of the increase and reduction of Nrf2 and Keap1, respectively, resulted in a substantial elevation of the Nrf2-to-Keap1 ratio, facilitating the nuclear translocation of Nrf2 and subsequent upregulation of the antioxidant enzyme Catalase, whose expression was associated to that of Nrf2. No association was observed between Nrf2 signalling and SOD1 and SOD2 protein expressions. While SOD2 did not change significantly during either exercise or ischaemia, SOD1 protein expression was downregulated and upregulated during exercise in normoxia and hypoxia, respectively (see Fig. 8 for a graphical summary).

Contrary to our hypothesis, the application of immediate ischaemia at exhaustion did not amplify the changes elicited by the bout of intense exercise until exhaustion in pThr^287^ CaMKII, pThr^172^ AMPKα, Nrf2 nor Keap1. Nonetheless, Catalase protein expression was further increased after the application of ischaemia at exhaustion in hypoxia. By using a novel experimental approach in humans, we have demonstrated that Nrf2, Keap1 and Catalase have a fast turnover in exercised human skeletal muscle, recovering pre-exercise levels within 1 min after the end of a bout of intense exercise by an O_2_-dependent mechanism. Interestingly, during recovery with free circulation after exhausting exercise in hypoxia, SOD1 increased while Catalase was reduced, revealing a different regulation of these two critical antioxidant enzymes in response to exercise.

### 5.1 Skeletal muscle Keap1 protein levels are reduced by intense exercise

The present investigation demonstrates for the first time that exhaustive exercise reduces the amount of Keap1 protein in human skeletal muscle. Keap1 is a substrate adaptor protein for the Cul3 RING-box 1 (RBX1) E3 ubiquitin ligase which ubiquitinates Nrf2 for proteasomal degradation in response to oxidants and electrophiles [[48], [49], [50]]. Heavy metals and several oxidative and electrophilic agents may induce thiol modifications in Keap1 which impair Nrf2 ubiquitination, resulting in Nrf2 protein accumulation [25]. These type of Keap1 modifications have been observed in cells treated with oxidized lipids [51], H_2_O_2_ [52], and nitric oxide [52,53].

The drop in Keap1 levels could be due to reduced synthesis of the protein, increased degradation, or both. Our results provide evidence for increased proteasomal and autophagic degradation of Keap1 during exercise, likely triggered by its oxidative modification by RONS [[1], [2], [3]]. Although oxidative stress markers were not directly assessed in this investigation, the increased Thr^287^ CaMKII phosphorylation supports this explanation [5]. Likewise, the observed augmented levels of Ser^40^ Nrf2 phosphorylation combined with the reduction of Keap1 levels and the negative association between Thr^287^ CaMKII phosphorylation and Keap1 are also compatible with increased RONS-mediated signalling.

### 5.2 Skeletal muscle total Nrf2 and its Ser^40^ phosphorylation are increased by exhaustive exercise in humans with similar responses in normoxia and severe acute hypoxia

Nrf2 abundance was elevated at exhaustion suggesting increased *de novo* synthesis or reduced proteasomal degradation during exercise. The observed activation of AMPKα may have prevented the degradation of Nrf2 by stimulating the p62-mediated autophagy of Keap1 [[54], [55], [56]] or by phosphorylating and inhibiting glycogen synthase kinase-3-β (GSK3-β), which phosphorylates and activates β-transducin repeat-containing E3 ubiquitin-protein ligase (β-TrCP) resulting in Nrf2 ubiquitination and proteasomal degradation [57,58]. In agreement with p62-mediated autophagy of Keap1 elicited by AMPK phosphorylation of its serine 349 ^56^, the fractional phosphorylation of p62 was associated with that of Thr^172^ AMPKα in the present investigation. Besides, p62 protein was significantly reduced after exercise, suggesting co-degradation with Keap1 [59]. The fact that Keap1 protein content was lowered supports a reduction of Nrf2 proteasomal degradation during intense exercise, as indicated by the inverse association between the two observed here. Nevertheless, the marked increase of the Nrf2-to-Keap1 ratio also indicates stimulation of Nrf2 *de novo* synthesis.

It must be emphasized that the level of hypoxia utilized here is close to the limit that humans can tolerate without altitude acclimatization [60]. RONS production in skeletal muscle is exacerbated by exercise in hypoxia, likely due to higher activation of the anaerobic metabolism [19]. In fact, we have previously shown increased protein carbonylation in human skeletal muscle during prolonged sprint exercise performed at this level of hypoxia [19]. Despite the latter, and in contrast with our hypothesis, Nrf2 accumulation and Keap1 reduction were similar at exhaustion in normoxia and hypoxia, indicating that the stimulation of Nrf2 signalling was already maximal in normoxia or that the additional reduction in cellular PO_2_ during exercise in hypoxia was not sufficient as to stimulate Nrf2 accumulation or Keap1 reduction further, perhaps counteracted by unknown mechanisms.

Serine^40^ phosphorylation of Nrf2 by the ROS-sensitive kinase PKCδ [27] is thought to facilitate its nuclear translocation [61] and gene transactivation [62], although experimental evidence is not conclusive [25]. The present investigation shows that pSer^40^ Nrf2 expression is increased during high-intensity exercise to a similar extent when the exercise is performed in normoxia and severe acute hypoxia. This may have facilitated nuclear translocation and gene transactivation, as supported by the increased protein expression of Catalase.

### 5.3 Catalase protein expression is increased during incremental exercise to exhaustion

No previous study has determined acute changes in Catalase protein expression with acute exercise in humans. The few studies measuring this protein in humans have focussed on basal levels, reporting either an increased expression of the protein [63] or no change [36]. Three days after a 20 min high-intensity intermittent exercise session, basal levels of Catalase and SOD2 protein expression were increased in the human *vastus lateralis* muscle [64]. Likewise, increased SOD2 mRNA expression has been reported in human skeletal muscle 3 h after high-intensity and prolonged continuous exercise for 50 min [17].

Catalase is localized principally in peroxisomes but is also present in mitochondria [65]. The fast increase in Catalase protein expression (within minutes of exercise and within seconds during ischaemia after the incremental exercise in hypoxia) is likely necessary to counteract an increased H_2_O_2_ produced during exercise and ischaemia. This experimental observation concurs with the proposed role of H_2_O_2_ as a crucial signal driving the skeletal muscle adaptations to exercise [66]. Interestingly, cardiomyocyte overexpression of either Catalase or SOD2 results in increased lethality when transgenic mice are submitted to a forced-swimming program [67]. In these mice, the overexpression of SOD2 increases H_2_O_2_ production exceeding the detoxifying capacity of Catalase and peroxidases during exercise, leading to pathological levels of oxidative stress [67]. Overexpression of Catalase may result in hampering of signalling events necessary for the normal adaptation to exercise [66], causing maladaptation and increased death in transgenic mice submitted to repeated forced swimming [67]. Here we have observed a transient increase of Catalase expression partly reverted within seconds after exercise and no significant changes of SOD2. This contrasts with the increased SOD2 protein content observed after endurance training in human skeletal muscle [36,68]. However, we cannot rule out a delayed SOD2 increase in our study since no additional muscle biopsies were performed to check for changes in the following hours after the exercise.

Thus, we have demonstrated that skeletal muscle can increase the amount of critical antioxidant enzymes acutely, likely via RONS-stimulated Nrf2 activation. When the exercise is stopped the excess antioxidant capacity build-up during the exercise is to a large extent, if not wholly, reversed to restore the redox balance to pre-exercise levels avoiding the risks of excessive reductive capacity [69].

### 5.4 Catalase and SOD1 are differentially regulated in response to exercise

Previous studies indicate that SOD1 is constitutively expressed with limited regulation by external stimuli [70]. SOD1 is located in the cytoplasm, nucleus and outer mitochondrial membrane, while Catalase is a predominantly extramitochondrial protein, but also found in the mitochondrial matrix [67,70]. SOD1 was slightly increased during exercise in hypoxia, facilitating the dismutation of superoxide generated by extramitochondrial oxidases [71]. This response concurs with the observed increased RONS production during high-intensity exercise in hypoxia [3], which has a significant cytoplasmatic component [66,71]. In the presence of higher levels of SOD1, the production of H_2_O_2_ is likely increased during exercise in severe hypoxia, requiring a higher amount of Catalase to avoid unchecked oxidative damage. In agreement with this explanation, it has been reported that H_2_O_2_ may induce SOD1 gene transcription by an Nrf2-independent mechanism [72]. Besides, it has been shown that H_2_O_2_ promotes SOD1 nuclear localization, where it acts as a transcription factor promoting the expression of antioxidant enzymes [73]. The remarkable acute increase of Catalase expression after exercise in hypoxia and during ischaemia is likely necessary to counteract an excessive H_2_O_2_ production during exercise and after the application of ischaemia [74,75]. The latter was accompanied by the expected, although non-statistically significant, changes in the protein expression of Ser^40^ Nrf2, Nrf2 total, Nrf2 total/Keap1, and Keap1. The fact that the antioxidant enzymes increased in response to exercise and ischaemia are located mostly outside the mitochondria is compatible with a predominant extramitochondrial production of RONS during exercise [71], which may be even more marked during exercise in hypoxia and ischaemia.

### 5.5 Keap1 levels recover rapidly after the cessation of contractile activity in an O_2_-dependent mechanism

In the present study, subjects performed exercise until their limit, and upon exhaustion, a pneumatic cuff was instantaneously inflated to 300 mmHg to fully occlude the circulation in less than 2 s in one leg, while the other leg recovered without circulatory restraints. During the first 3–5 s of the occlusion, the O_2_ stores (O_2_ trapped in capillary blood and bound to myoglobin) are depleted by oxidative phosphorylation, which is strongly stimulated [2,42]. This was evidenced by the fast reduction and plateauing of muscle oxygenation measured by near-infrared spectroscopy (NIRS), as previously reported [42]. The first post-exercise muscle biopsy was obtained 10 s after the end of the exercise, preventing potential effects of early oxygenation at exhaustion on muscle signalling. Then, the leg biopsied first remained occluded and, after 60 s another two muscle biopsies were obtained from the occluded and non-occluded leg simultaneously. This allowed a direct comparison of the occluded and non-occluded leg. During the 60 s of occlusion, the energy metabolism remained active in the occluded leg, utilizing the energy supplied by the glycolysis, leading to a higher accumulation of lactate, H^+^, Pi and Cr. In contrast, the concentration of ATP remained at the same level reached at exhaustion, i.e. ~80% of the concentration observed before exercise [42]. Despite the increased build-up of glycolytic metabolites during the 60 s occlusion, no further increase of Nrf2 or reduction of Keap1 was detected in the occluded leg. Although we cannot rule out some RONS production from 10 to the 60 s of ischaemia [74,75], its magnitude should have been small as indirectly indicated by stability during this period of both pThr^287^ CaMKII, pSer^40^ Nrf2 and Keap1, which are sensitive to RONS.

In the leg recovering with free circulation, Nrf2, Keap1 and the Nrf2-to-Keap1 ratio returned to pre-exercise levels within 60 s after the end of exercise, even though muscle lactate and H^+^ remained at the same level reached at exhaustion [42]. In contrast, Pi and Cr were reduced, and PCr increased during the 60 s of recovery with free circulation, without reaching pre-exercise values. This also indicates that the glycolytic metabolites accumulated during exercise do not play an essential role in either eliciting or maintaining Nrf2 activation. Nevertheless, the massive increase of Pi, which led to almost depletion of PCr during ischaemia may have inhibited the phosphatases [29], keeping the phosphorylation levels during ischaemia.

The principal difference between ischaemic and free circulation recoveries is the presence of O_2_. Femoral vein PO_2_, a surrogate of mean capillary PO_2_, is rapidly increased after the cessation of contractile activity as it was reflected by the NIRS signal captured at the end of exercise in the perfused leg [42]. The production of ATP by oxidative phosphorylation is likely mandatory to reactivate the *de novo* synthesis of Keap1 in skeletal muscle. Despite the shortage of energy during ischaemia, mainly when applied at exhaustion following exercise in severe acute hypoxia, Catalase protein content was almost doubled after the IE in Hyp, indicating, that even in ischaemia the synthesis of some proteins is still active [76]. The reason why Nrf2 levels were not reduced to pre-exercise values during ischaemia may be, in part, explained by the attenuation of global protein degradation in anoxia [77]. In the leg recovering with free circulation, Nrf2 was reduced to the pre-exercise level already after only 1 min, likely through proteasomal and autophagy degradation facilitated by the fast increase of Keap1 during recovery.

It has been suggested that once modified by oxidants or electrophiles, Keap1 is committed to p62-mediated autophagy [59]. Keap1 gene harbours an ARE promoter which may be stimulated by Nrf2 to enhance its new translation [25,78] immediately after exercise. Besides, part of the oxidized Keap1 may have been regenerated by thioredoxin reductase 1 [25] during the 1-min recovery with open circulation. It remains to be determined whether the application of post-exercise ischaemia could be used to enhance Nrf2-mediated adaptation, as observed in tissues submitted to repeated episodes of ischaemia-reperfusion [76,79].

### 5.6 Practical implications

The current experiments have two practical implications. First, we have demonstrated that some signalling events triggered by exercise recovered with rather fast kinetics. The importance of obtaining fast muscle biopsies during exercise was already pointed out by exercise physiologists in the 70–80s to capture metabolic changes with rapid recovery kinetics [80,81]. Secondly, we have shown, as a novelty, that post-exercise ischaemia allows maintaining the end exercise metabolic conditions during the first seconds of recovery, facilitating the capture of signals that otherwise will escape detection.

### 5.7 Limitations

In the present research, subjects were free to ingest their usual diet. Some components of the food may influence Nrf2 basal levels [82] and perhaps Nrf2 signalling responses to exercise. Although we asked our subjects to maintain their habitual diet the week before an experiment and reproduce the same dinner the day before the two experimental conditions, we cannot rule out a potential confounding effect of small differences in the composition of the diet. Nevertheless, the fact that the basal expression of Nrf2 and antioxidant enzymes were similar between the two conditions indicates that experiments started with comparable basal conditions.

In the present investigation rather than measuring oxidative stress markers, we focused on assessing signalling events that are predominantly elicited by RONS. Although this approach is more sensitive to changes in RONS, it is also less specific, because both CaMKII and AMPK may be activated by mechanisms independent from RONS [37,83]. However, the reduction of Keap1 seems more specific as a marker of increased production of RONS [52,84]. Another limitation of the present experiments is that our findings are limited to the antioxidant enzymes assessed, i.e., catalase, SOD1 and SOD2 and to the time frame of our experiments.

In summary, this study shows that, during incremental exercise to exhaustion, Nrf2 signalling is upregulated to a similar extent in normoxia and severe acute hypoxia, by a mechanism connected to the decrease in Keap1 protein that promotes a remarkable elevation of Catalase protein content. These changes are almost entirely reverted to pre-exercise levels in less than 60 s by an O_2_-dependent mechanism. We have also shown that SOD1 protein content is differentially regulated in response to acute exercise to exhaustion in normoxia and hypoxia. Finally, we have demonstrated the importance of obtaining the muscle biopsies as close as possible to the end of exercise and the utility of post-exercise ischaemia to capture these swiftly-responding signals.

## Disclosure summary

The authors have nothing to disclose.

## Declaration of competing interest

The authors declare that they have no known competing financial interests or personal relationships that could have appeared to influence the work reported in this paper.
