# Supplementary material for: Regulation of Nrf2/Keap1 signalling in human skeletal muscle during exercise to exhaustion in normoxia, severe acute hypoxia and post-exercise ischaemia: Influence of metabolite accumulation and oxygenation
Source: Redox Biol. 2020 Jun 30;36:101627. doi: 10.1016/j.redox.2020.101627 (PMC7358388; doi:10.1016/j.redox.2020.101627)
Supplement: Multimedia component 1 [file mmc1.docx]

**Supplementary Table 1: Detailed description of Western blotting antibodies and procedures.**

| **Antibody** |  | **Manufacturer company** |  | **Catalog number** |  | **Protein**  **molecular weight (kDa)** |  | **Gel %** |  | **Protein amount loaded**  **(μg)** |  | **Electro-**  **phoresis running**  **time (min)** |  | **Blotting transfer time (min)** |  | **Blocking reagent** |  | **Primary antibody concentration** |  | **Secondary**  **antibody concentration** |
| --- | --- | --- | --- | --- | --- | --- | --- | --- | --- | --- | --- | --- | --- | --- | --- | --- | --- | --- | --- | --- |
| Thr^287^  CamKII |  | Cell Signaling |  | 12716 |  | 50-75 |  | 10 |  | 20 |  | 98 |  | 90 |  | BSA 4% |  | 1:2000 |  | 1:5000 |
| Thr^172^  AMPK |  | Cell Signaling |  | 2535 |  | 62 |  | 10 |  | 25 |  | 100 |  | 90 |  | BSA 4% |  | 1:2000 |  | 1:5000 |
| AMPKα |  | Cell Signaling |  | 2532 |  | 62 |  | 10 |  | 25 |  | 100 |  | 90 |  | BSA 4% |  | 1:2000 |  | 1:5000 |
| Ser^349^ SQSTM1/p62 |  | Abcam |  | ab211324 |  | 62 |  | 10-12.5 |  | 20 |  | 90 |  | 90 |  | BSA 4% |  | 1:2000 |  | 1:5000 |
| SQSTM1/p62 |  | Abcam |  | ab56416 |  | 62 |  | 10 |  | 25 |  | 40 |  | 90 |  | Blotto 5% |  | 1:3000 |  | 1:5000 |
| Catalase |  | Cell Signaling |  | 14097 |  | 60 |  | 10 |  | 10 |  | 120 |  | 90 |  | Blotto 5% |  | 1:2000 |  | 1:5000 |
| SOD1 |  | Abcam |  | ab16831 |  | 17 |  | 10-12.5 |  | 20 |  | 90 |  | 90 |  | BSA 4% |  | 1:2000 |  | 1:5000 |
| SOD2 |  | Cell Signaling |  | 13141 |  | 22 |  | 10-12.5 |  | 20 |  | 90 |  | 90 |  | BSA 4% |  | 1:20000 |  | 1:5000 |
| Ser^40^  Nrf2 |  | Abcam |  | ab76026 |  | 100 |  | 4-20% |  | 15 |  | 75 |  | 90 |  | BSA 4% |  | 1:5000 |  | 1:5000 |
| Nrf2 |  | Abcam |  | ab62352 |  | 100 |  | 10 |  | 15 |  | 75 |  | 90 |  | BSA 4% |  | 1:500 |  | 1:5000 |
| Keap1 |  | Abcam |  | ab119403 |  | 70 |  | 4-20% |  | 15 |  | 75 |  | 90 |  | BSA 4% |  | 1:2000 |  | 1:5000 |
